# Epidemiological Comparison of Four COVID-19 Waves in the Democratic Republic of the Congo, March 2020–January 2022

**DOI:** 10.1007/s44197-022-00052-6

**Published:** 2022-08-03

**Authors:** John Otokoye Otshudiema, Gervais Léon T. Folefack, Justus M. Nsio, Placide Mbala-Kingebeni, Cathy H. Kakema, Joel B. Kosianza, Antoine K. Mfumu, Guy N. Saidi, Patrice M. Kabongo, Raphael Okum, Tshibambe N. Tshimbombu, Steve Ahuka-Mundeke, Humphrey Cyprian Karamagi, Jean-Jacques T. Muyembe, Amédée Prosper Djiguimde

**Affiliations:** 1 COVID-19 Incident Management System, Health Emergencies Program, World Health Organization, Kinshasa, Democratic Republic of the Congo; 2 COVID-19 Response, Epidemiological Surveillance Directorate, Ministry of Health, Kinshasa, Democratic Republic of the Congo; 3 COVID-19 Response, Department of Medical Microbiology and Virology, Faculty of Medicine, University of Kinshasa, National Institute of Biomedical Research, Kinshasa, Democratic Republic of the Congo; 4 Dartmouth Geisel School of Medicine, Hanover, NH USA; 5 COVID-19 Incident Management System, Data Analytics and Knowledge Management, World Health Organization Regional Office for Africa, Brazzaville, Republic of Congo

**Keywords:** COVID-19, Democratic Republic of the Congo, Epidemiological comparison, Resurgence, Wave

## Abstract

**Purpose:**

Nationwide analyses are required to optimise and tailor activities to control future COVID-19 waves of resurgence continent-wide. We compared epidemiological and clinical outcomes of the four COVID-19 waves in the Democratic Republic of Congo (DRC).

**Methods:**

This retrospective descriptive epidemiological analysis included data from the national line list of confirmed COVID-19 cases in all provinces for all waves between 9 March 2020 and 2 January 2022. Descriptive statistical measures (frequencies, percentages, case fatality rates [CFR], test positivity rates [TPR], and characteristics) were compared using chi-squared or the Fisher–Irwin test.

**Results:**

During the study period, 72,108/445,084 (16.2%) tests were positive, with 9,641/56,637 (17.0%), 16,643/66,560 (25.0%), 24,172/157,945 (15.3%), and 21,652/163,942 (13.2%) cases during the first, second, third, and fourth waves, respectively. TPR significantly decreased from 17.0% in the first wave to 13.2% in the fourth wave as did infection of frontline health workers (5.2% vs. 0.9%). CFR decreased from 5.1 to 0.9% from the first to fourth wave. No sex- or age-related differences in distributions across different waves were observed. The majority of cases were asymptomatic in the first (73.1%) and second (86.6%) waves, in contrast to that in the third (11.1%) and fourth (31.3%) waves.

**Conclusion:**

Despite fewer reported cases, the primary waves (first and second) of the COVID-19 pandemic in the DRC were more severe than the third and fourth waves, with each wave being associated with a new SARS-CoV-2 variant. Tailored public health and social measures, and resurgence monitoring are needed to control future waves of COVID-19.

## Introduction

The coronavirus disease 2019 (COVID-19) is an infectious disease that is caused by the severe acute respiratory syndrome coronavirus 2 (SARS-CoV-2). The first cases were officially recorded on 31 December 2019 in the Chinese city Wuhan, China. The virus is spreading rapidly and outbreaks can grow exponentially [1, 2]. COVID-19 was officially categorized by the World Health Organization (WHO) as a public health emergency of international concern on 30 January 2020 and, on 11 March 2020, as a pandemic [3, 4]. By 3 July 2022, the number of the reported COVID-19-related deaths was past 6.3 million worldwide [5], with disease propagation occurring in waves of resurgence [6–8]. The first imported case of COVID-19 in Africa was reported from Egypt on 14 February 2020, and the pandemic has subsequently affected, to various degrees, all countries in the African continent. On 14 December 2021, the fourth wave of the COVID-19 pandemic, which was predominantly stimulated by the Omicron subtype, officially entered Africa [9–11]. Available event-based aggregate data show variations in the characteristics of the first and second waves, with heterogeneity within the five regions and 55 countries [12].

In the Democratic Republic of Congo (DRC), the first COVID-19 case, involving an individual traveling from Europe was recorded on 10 March 2020. By 2 January 2022, there were 80,562 confirmed COVID-19 cases in the DRC. The DRC boosted their SARS-CoV-2 testing capacity by increasing the number of laboratories from 1 to 25 through decentralisation of coronavirus polymerase chain reaction (PCR) testing as well as using SARS-CoV-2 antigen rapid diagnostic tests (Ag-RDT). The COVID-19 Vaccines Global Access (COVAX) initiative supplied the first batch of COVID-19 vaccines to the DRC in April 2021 [13]. However, as of 3 July 2022, only 4% of the total population had been fully vaccinated [14], which is one of the lowest coverage rates worldwide. The DRC is experiencing widespread community-based SARS-CoV-2 transmission and has already experienced four waves of resurgence.

Currently, there are several official epidemiological definitions of the COVID-19 waves. One of the main characteristics of a wave is an increasing number of registered cases, followed by a clear and identifiable peak and a subsequent decline [15–18]. However, more precise definitions are needed, as epidemiological and policy actions differ according to the state of the pandemic. In this study, we aimed to information obtained from the four described COVID-19 waves in the DRC to determine the epidemiological features and clinical outcomes of the COVID-19 waves in the DRC.

## Methods

### Design, Timeline, and Preparation

This retrospective descriptive study using epidemiological data from the national line listing of COVID-19 laboratory-confirmed cases, analysed the data of cases reported in 26 provinces of the DRC that were registered between 9 March 2020 and 2 January 2022, and were distributed by epidemiological weeks (EW). The study period was stratified according to the EW of notification for each phase, to identify temporal trends in cases as well as waves of resurgence, as defined by the WHO. Additional data from laboratory and medical case management databases were analysed. Provincial databases were compiled at the national level by the National Multi-sectorial Committee of the COVID-19 Response (CMR COVID-19). Information on SARS-CoV-2 variants associated with a specific wave was obtained from DRC’s National Institute of Biomedical Research. We used the available online climate data from *Climate-Data.org* [19] to compare the COVID-19 waves based on the seasonal dynamics.

### Data Collection and Study Population Selection

The study population comprised those who had positive test results for SARS-CoV-2 infection and whose records had been entered into the national line listing by wave category at the time of study completion (2 January 2022). The inclusion of cases was rationalised primarily by defining the case category of a suspected COVID-19 case, which has been defined in the DRC in two ways: (1) any person who presents with one or more sign(s) or symptom(s) of acute respiratory infection, regardless of disease severity; and (2) any person who was in close contact (high-risk contact) with a confirmed case and who did or did not have one or more signs or symptoms of COVID-19. Furthermore, testing was performed for the population groups residing and working in high-risk environments containing infection sources, including those in close contact with a confirmed positive case, healthcare employees or frontline workers, community participants, and workers in closed spaces (e.g., prisoners), participants of a mass gathering, and incoming or outgoing travellers, as part of mandatory screening at all points of entry and borders.

Each participant who underwent the test procedures received a special case-investigation questionnaire form to collect socio-demographic data as well as travel and clinical histories. The procedures for sample collection, packaging, and delivery to a laboratory setting followed the national COVID-19 response guidelines and protocols. At least one nasopharyngeal and oropharyngeal swab, based on synthetic fibres equipped with plastic shafts, had been obtained. COVID-19 confirmatory diagnostics included PCR as the gold standard, along with the Ag-RDT kit. The PCR specimens were triple-packed and transported in a sterile container, whilst using special viral tools and conditions for secure transportation and maintaining adequate and stable temperatures (2–4 °C). A confirmed case is any individual with a laboratory result (PCR or Ag-RDT) that confirms SARS-CoV-2 infection, regardless of overt or covert COVID-19.

### Definition of a COVID-19 Resurgence and Wave

According to the WHO, the COVID-19 resurgence represents a visible growth in new COVID-19 cases that are registered following at least two consecutive weeks of low or no transmission, assuming optimal surveillance and testing activities [20]. The COVID-19 wave is a situation wherein a sudden increase beyond the expected number of COVID-19 cases is observed and is scientifically defined based on the shape of the epidemic curve, which requires mathematical calculations [21]. The two main actionable thresholds that define the start and end of a wave of COVID-19 transmission are the resurgence response and the ‘under control’ phase, respectively. The COVID-19 resurgence-response threshold is defined as an increase of at least 20% incident cases in the preceding 2 weeks (using a 7-day moving average) or a sudden increase in cases, which exceeds the previous peak by 30%. Consequently, SARS-CoV-2 transmission is assumed to be controlled when the increase in incident cases in terms of the 7-day moving average is less than 10% for two consecutive weeks, or a steady reduction or an epidemiological plateau over two consecutive weeks is registered. Additionally, the threshold of a resurgence alert is achieved when a 10–20% increase in the number of confirmed COVID-19 cases has been recorded (the 7-day moving average). For a geographical area to be classified as under control, none of the criteria for a resurgence alert or response should be present [20, 21]. Achieving the peak of the pandemic curve implies that the numbers of newly registered cases start to align gradually instead of moving upward on an abrupt trajectory.

### Definition of Variables

In this study, the unit of analysis was the confirmed COVID-19 case reported for each wave. The description of the key characteristics for each wave included the duration of waves and transition periods, number of cases and deaths, epidemic peaks, variants, age, sex, symptomatic case status, travel history, and infection status of frontline health workers. In the DRC, the following parameters were compared between the four COVID-19 waves: case fatality rate (CFR), test positivity rate (TPR), percentage (%) of cases with travel history, and percentage (%) of infected frontline healthcare workers. The principal outcomes of this research project have been associated with CFR and TPR, which was the proportion (%) of all tests, completed that showed positive outcomes for SARS-CoV-2, as determined below:

$$x = \frac{a \times 100}{b}$$

where *x*, the TPR; *a*, the number of confirmed COVID-19 cases during the study period; and *b*, the overall number of COVID-19 tests completed during the study period.

The CFR is the proportion of individuals diagnosed with COVID-19 who died during the study period. The median age was determined for confirmed cases and deaths due to COVID-19. Depending on the travel history and provenance of cases among travellers, we classified the confirmed COVID-19 cases as local or imported cases (global travel history during the preceding 2 weeks). Individuals manifesting at least one symptom were marked as symptomatic, whereas individuals without any symptoms (such as fever, cough, headache, shortness of breath, anosmia, ageusia, and malaise) during tests were marked as asymptomatic. A new variant was identified in each wave. Furthermore, we compared seasonal parameters, including temperature and rainfall over time and the number of confirmed cases in the Kinshasa main hotspot.

### Statistical Analysis

Statistical analysis was performed using R version 4.0.5 (The R Project for Statistical Computing 4.0.5. GNU GPL v2) [22]. Graphs were created using Microsoft Excel 2010. The results are presented in summary tables and epidemic curves using weekly cumulative cases to demonstrate the time trend. Epidemiological and clinical data of confirmed COVID-19 cases were compared between the different waves. Numbers (proportions) and median values (minimum and maximum) or mean values (standard deviation) are reported for categorical and continuous variables, respectively. Participant characteristics were compared using descriptive statistical methods. For categorical data, the chi-squared or Fisher–Irwin tests and the significance level were used, as appropriate, for a comparison of proportions using the calculator of MedCalc Software Ltd Version 20.027 and applying the "N-1" chi-squared test following the recommendations from previous studies, such as Richardson (2011) and Campbell (2007) [23, 24]. A *P* value less than 0.05 was considered indicative of statistical importance.

## Results

### Defining COVID-19 Waves in the DRC

**Fig. 1:**
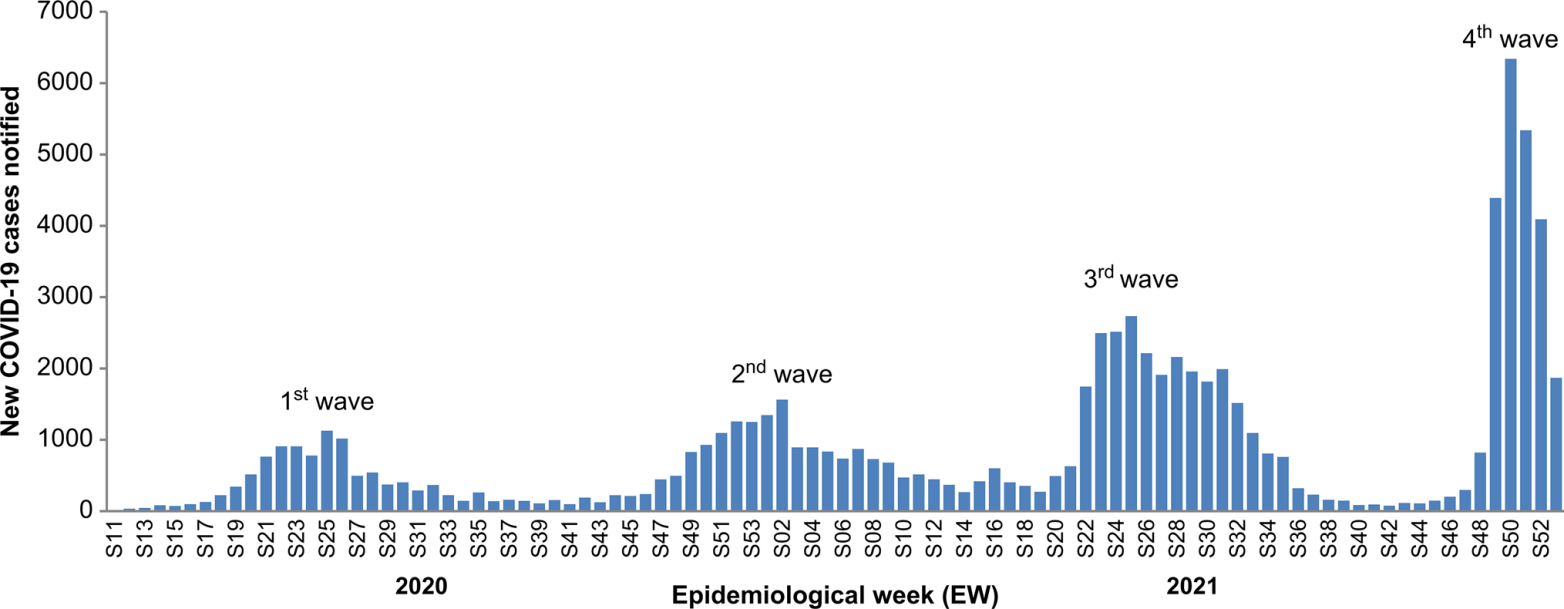
Epidemic curve of confirmed COVID-19 cases reported by epidemiological week (EW) and by defined wave with related epidemiological characteristics in the DRC from EW11-2020 to EW52-2021

According to the WHO’s definition of COVID-19 waves, from 9 March 2020 through 2 January 2022, the available EW-wise aggregated data facilitated the identification of eight phases of the epidemic, including four waves and four under-control and alert periods (Fig. 1):

- Phase I: 03/09/2020–04/12/2020 (EW11/2020–EW15/2020)
- Phase II: 04/13/2020–08/23/2020 (EW16/2020–EW34/2020) = first wave
- Phase III: 08/24/2020–11/15/2020 (EW35/2020–EW46/2020)
- Phase IV: 11/16/2020–04/04/2021 (EW47/2020–EW13/2021) = second wave
- Phase V: 04/05/2021–05/16/2021 (EW14/2021–EW19/2021)
- Phase VI: 05/17/2021–08/15/2021(EW20/2021–EW32/2021) = third wave
- Phase VII: 08/16/2021–11/07/2021 (EW33/2021–EW44/2021)
- Phase VIII: 11/08/2021–01/02/2022 (EW45/2021–EW52/2021) = fourth wave

### Duration of Waves and Transition Periods

The first wave occurred 5 weeks after the first registered confirmed case and lasted for 19 weeks. The second wave occurred 12 weeks after the first (Fig. 1 and Table 1). The third wave lasted 13 weeks and occurred 6 weeks after the second wave, whereas the fourth wave occurred 12 weeks after the third wave and lasted only 8 weeks (Fig. 1 and Table 1). The mean duration of the COVID-19 waves was 14.7 (SD 5.3) weeks whereas the transition periods (under control and alerts) between the waves lasted 8.7 (SD 3.7) weeks.

**Table 1:** Characteristics of peak transmission during the four waves of COVID-19 in the DRC, March 2020–January 2022

	First wave	Second wave	Third wave	Fourth wave
Wave, EW^a^	EW16–EW34/2020	EW47/2020–EW13/2021	EW20–EW32/ 2021	EW45–EW52/2021
Wave, period	13 Apr–23 Aug 2020	16 Nov 2020–04 Apr 2021	17 May–15 Aug 2021	08 Nov 2021–02 Jan 2022
Peak, EW^a^	EW25	EW02	EW25	EW50
Peak, period	15–21 Jun 2020	11–17 Jan 2021	21–27 Jun 2021	13–19 Dec 2021
# of cases during the peak’s EW^a^	1128	1563	2734	6341
% of increase		39% (1 vs. 2)	75% (2 vs. 3)	132% (3 vs. 4)

^a^EW, epidemiological week

Four peaks of transmission related to the four waves of COVID-19 resurgence in the DRC occurred from 9 March 2020 through 2 January 2022 (96-week period or approximately 22-month period; Table 1). The first and third waves occurred during the same period: 15–21 June 2020 and 21–27 June 2021, respectively. The second and fourth waves occurred in the same period: 11–17 January 2021 and 13–19 December 2021, respectively.

The most important peak occurred during the fourth wave, with 6,341 cases observed during the EW50, which corresponded to a 132% growth in the number of cases compared to the peak value during the third wave (Table 1) that was characterised by an increase in the number of new cases that were associated with the circulation of the Delta variant, which was officially detected in the DRC in June 2021. The fourth wave was associated with the Omicron variant, which was officially confirmed in the DRC on 13 December 2021.

### Numbers of Cases and Deaths During the Four Waves of COVID-19 in the DRC

During the first wave (13 April 2020–23 August 2020), 56,637 screening tests confirmed 9,641 documented cases (TPR: 17.0%), and 496 deaths were recorded (CFR: 5.1%; Table 2). During the second wave (16 November 2020–04 April 2021), the number of screening tests increased to 66,560 cases, and confirmed 16,643 cases (TPR: 25.0%) with nearly 400 recorded deaths (CFR: 2.4%; Table 2). The third wave (17 May 2021–15 August 2021) was characterised by a higher number of screening tests (157,945), incident COVID-19 cases (24,172), and deaths (570); the TPR was 15.3% (CFR: 2.1%). During the fourth wave (08 November 2021–02 January 2022), 163,942 screening tests confirmed 21,652 cases (TPR: 13.2%) and there were 204 recorded deaths (CFR: 0.9%; Table 2).

**Table 2:** Comparison of epidemiological and clinical outcomes during the four waves of the COVID-19 pandemic in the DRC, from 9 March 2020 to 2 January 2022

Characteristics	First wave	Second wave	Third wave	Fourth wave	*P *value					
	(EW16 –EW34, 2020)	(EW47, 2020–EW13, 2021)	(EW20–EW32, 2021)	(EW45–EW52, 2021)	(1 vs. 2)	(1 vs. 3)	(1 vs. 4)	(2 vs. 3)	(2 vs. 4)	(3 vs. 4)
Epidemiological characteristics										
Epidemiological week	19	19	13	8						
Confirmatory tests performed	56,637	66,560	157, 945	163,942						
Confirmed cases	9641	16,643	24,172	21,652						
Test positivity rate	17.0%	25.0%	15.3%	13.2%	<0.0001	<0.0001	<0.0001	** < 0.0001**	** < 0.0001**	<0.0001
Deaths	496	399	570	204						
Case fatality rate	5.1%	2.4%	2.4%	0.9%	0.0381	0.0192	0.0089	1.0000	0.2013	0.1899
Age, years, median (min–max)										
Cases	41 (1**–**98)	40 (1**–**100)	40 (1**–**107)	38 (1**–**98)						
Deaths	60 (1**–**98)	65 (7**–**100)	65 (2**–**91)	60.5 (20**–**80)						
Sex, male, *N* (%)	6393 (66.3%)	10,181 (61.2%)	15,047 (62.2%)	13,112 (60.6%)	<0.0001	<0.0001	<0.0001	0.1088	0.3520	0.00559
Asymptomatic cases, *N* (%)	2372/3246 (73.1%)	3963/4577 (86.6%)	146/1321 (11.1%)	309/987 (31.3%)	<0.0001	<0.0001	<0.0001	<0.0001	<0.0001	<0.0001
Cases with travel history (imported cases), *N* (%)	52 (0.5%)	488 (2.9%)	1093 (4.5%)	284 (1.3%)	0.3078	0.1658	0.6233	0.1341	0.1539	0.0124
Infected frontline health workers, *N* (%)	497 (5.2%)	185 (1.1%)	189 (0.8%)	190 (0.9%)	0.0163	0.0086	0.0102	0.7650	0.8458	0.9157

A *P*-value <0.0001 (bold) means that the difference was statistically significant or relevant

The number of screening tests increased from 56,637 during the first wave of coronavirus to 66,560 in the second wave. During the third wave, the numbers were 157,945, followed by 163,942 in the fourth wave, representing an 18% (first vs. second wave), 137% (second vs. third wave), and 4% (third vs. fourth wave) increase, respectively (Table 2). A statistically relevant (*P* < 0.001) growth in the proportion of screening TPR and asymptomatic status during the four  waves (Table 2), was identified.

### Social and Demographic Parameters

The social and demographic parameters of the confirmed COVID-19 cases and deaths, distributed by wave categories, are summarised in Table 2. The median (min–max) age of confirmed cases, approximately 40 (1,107) years, was lower than that of deaths, approximately 63 (1, 100) years (Table 2). The median age of cases and deaths was similar in the four waves. The proportion of women—approximately 37%—was similar in the four waves. The proportion of infected frontline healthcare workers decreased over time from 5.2% (497/9641) in the first wave to 0.9% (190/21,652) in the fourth wave (Table 2). However, the differences between these proportions were not statistically significant. The proportion of individuals with positive testing outcomes at the point of entry among travellers and/or those who had records of travelling in a 2-week period (imported cases) was similar in the four waves (Table 2).

### Symptoms

The proportion of confirmed cases characterised as asymptomatic was higher (86.6%) during the second wave in contrast to the first (73.1%), the third (11.1%), and the fourth (31.3%) waves. This decrease, from the second to the third and fourth waves, was statistically significant (Table 2).

### Main Hotspot

The Kinshasa Province and the capital of DRC were consistently the most-affected provinces in the four waves in terms of the proportion of confirmed cases: 79.5%, 68.9%, 51.6%, and 52.2%, respectively (Table 3).

**Table 3:** Top three most-affected provinces or hotspots based on the number of confirmed COVID-19 cases in each wave in the DRC from EW11, 2020 to EW 52, 2021

First wave (*n* = 9641), *N* (%)	Second wave (*n* = 16,643), *N* (%)	Third wave (*n* = 24,172), *N* (%)	Fourth wave (*n* = 21,662), *N* (%)
Kinshasa 7,666 (79.5%) Nord Kivu 640 (6.6%) Kongo Central 426 (4.4%)	Kinshasa 11,467 (68.9%) Haut Katanga 1474 (9.8%) Kongo Central 1123 (6.7%)	Kinshasa 12,489 (51.6%) Nord Kivu 3029 (12.5%) Haut Katanga 1796 (7.4%)	Kinshasa 11,227 (52.2%) Kongo Central 2058 (9.5%) Haut Katanga 1846 (8.6%)

### Climate Characteristics of Kinshasa, the Main Hotspot

The DRC is characterised by diverse climates (equatorial, humid tropical, and tropical with a prolonged dry season, and coastal), which provide the country with abundant rainfall and sunshine. The difference in rainfall between the driest and wettest periods was 191 mm. The medium temperature throughout the year differed by 1.7 °C. December was identified as the month with the highest level of relative humidity (85.21%), whereas the month demonstrating the least relative humidity was August (65.26%). November had the largest number of rainy days (25.37 days), whereas July has the smallest number of rainy days (0.27 days). The lowest medium rainfall value of only 1 mm was recorded in July. November had the highest annual rainfall, with an average of 192 mm [19].

**Fig. 2:**
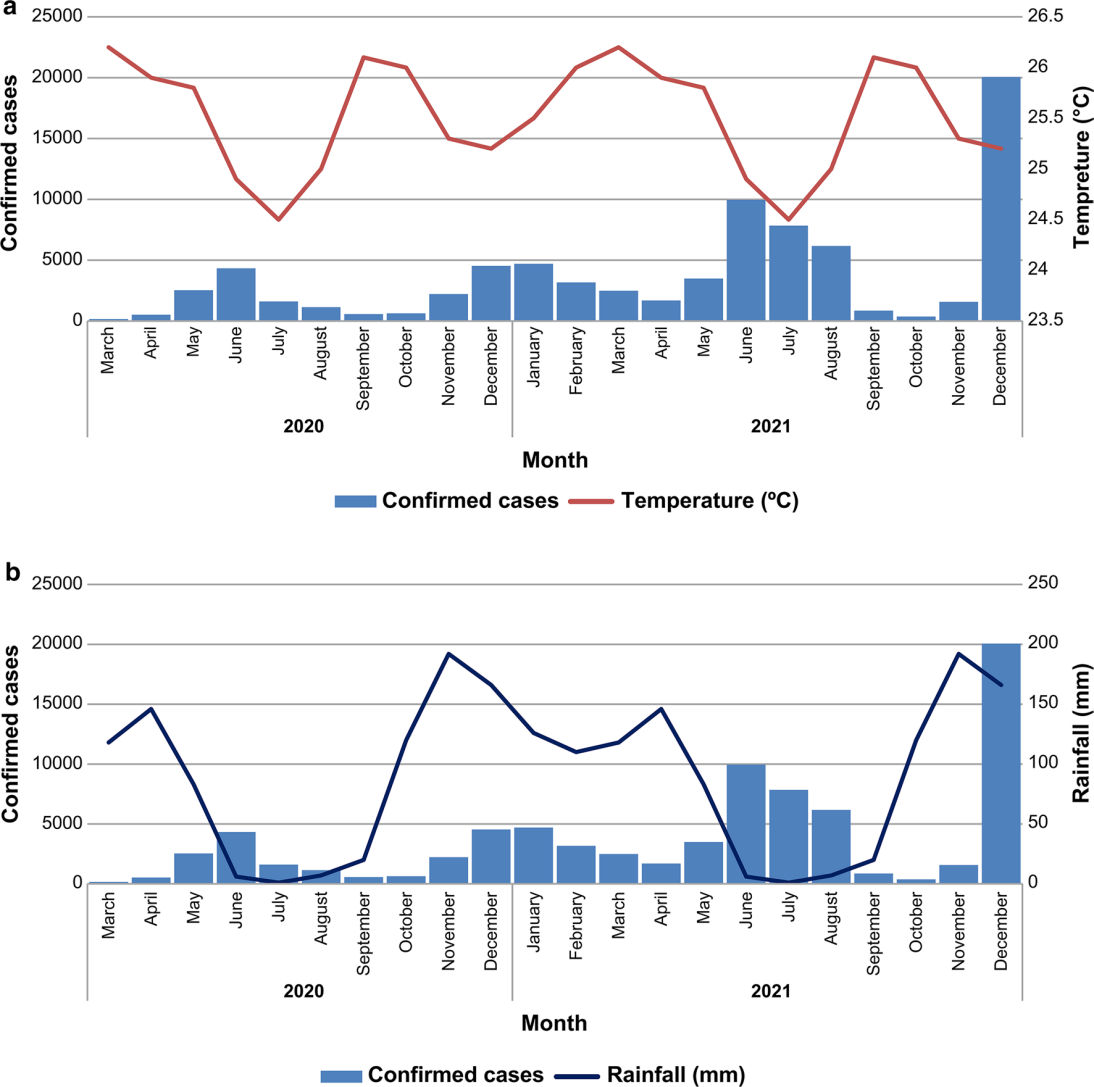
**a** Number of confirmed COVID-19 cases over time and temperature in Kinshasa. **b** Number of confirmed COVID-19 cases over time and rainfall in Kinshasa

The number of confirmed cases increased with low temperatures, with peaks occurring at periods of low temperatures and immediately after the peak rainfall periods (Fig. 2a and b). At least two peaks of transmission occurred during the year; the first peak was observed in mid-June and the second peak was observed between December and January of the following year (Fig. 2a and b).

## Discussion

To the best of our knowledge, this is the first study to describe and compare the epidemiological and clinical outcomes of the four waves of the COVID-19 resurgence in the DRC, including nationwide data. The word “wave” is attributed to a phenomenon characterised by drastic peaks and gradual declines. This suggests that, even in times of slowdown, there is potential for disease-related waves in the future [16–18, 20]. Three drivers of a surge in COVID-19 cases that cause a wave of COVID-19 are usually described: (a) a new population that has not been infected in the past, (b) a new variant with a different level of infectiousness or (c) reinfections among people who were previously infected [20]. There were no significant differences in the demographics across the four waves. The sex-ratio remained constant, with the proportion of males averaging 61% across all waves. Males were more affected, and this corroborates with growing evidence suggesting that males are more susceptible to infection and have higher morbidity and mortality from COVID-19 than females [25–27]. The median age of the confirmed cases was 40 years from the first to the fourth waves. However, the median age at death was elevated (60 years), even though it was similar across all four waves. The higher mortality in this age group may be due to their advanced age and the possible existence of comorbidities which predispose them to more severe disease [28]. The vaccination coverage in the DRC seems very low to drastically decrease the community transmission. A study on the association between the intensity and duration of non-pharmacological interventions (NPI) and implementation of COVID-19 vaccination using data from 22 European countries reported no significant added effects of vaccination on the growth rate of COVID-19 cases when the vaccination coverage rate was low (0%, 10%, and 20%) [29]. However, when a low vaccine coverage rate was coupled with implementation of strict NPI, the results from the same study revealed a significant effect. However, a population-based online survey that was conducted in 22 provinces of the DRC between 23 April and 8 June 2020 found that despite the imposition of compulsory restrictions by the government, only approximately 50% of the respondents adhered to COVID-19 preventive measures [30].

The proportion of infected frontline workers significantly decreased from the first to the fourth wave. Increased availability of personal protective equipment in health facilities across the nation, increased awareness of the fatality of the disease, COVID-19 vaccination, and increasing levels of natural immunity following infection are possible reasons for this observation [15]. This pattern of reduction in TPR was statistically significant in the second wave (25.0%), in contrast to that of the third (15.3%) and 4th (13.2%) waves. This is similar to the results obtained by Akande et al. [15] in Nigeria on comparing their first and second waves. The pattern also occurred with reduction in the attack rate and lower test positivity rate in later waves. However, the height of the peak of each wave increased from the first to the fourth wave. This could be explained by the occurrence of variants with high transmissibility in the population, specifically with the Delta and Omicron variants that were first isolated just prior to the third and fourth waves, respectively. This situation could have also been exacerbated by the reduction in adherence to non-pharmacological public health measures by the population as the pandemic progressed, due to fatigue. The asymptomatic status was statistically relevant during the four waves.

Detailed SARS-CoV-2 whole-genome sequencing data from DRC’s National Institute of Biomedical Research, showed that subsequent waves were associated with new variants. In some cases, some variants were more virulent, such as the Delta variant during the third wave, while a variant such as the Omicron variant during the fourth wave was less virulent but with a greater transmissibility due to its ability to evade immunity—whether naturally acquired or through vaccination [31]. The COVID-19 vaccine coverage after the second wave and different incriminating variants across the waves could explain why the third and fourth waves were shorter, lasting 13 and 8 weeks, respectively, compared to the first and second, with each lasting for 19 weeks. Another plausible theory is that the COVID-19 response improved over time in the DRC with an increase in the capacity of frontline responders, mobilisation of more resources and tools for intervention (including improved testing leading to early diagnosis and case management), coupled with a more informed and cooperative population following continuous sensitisation campaigns.

An 18% increase in the number of screening tests performed between the first and the second waves was observed, representing 137% (second vs. third wave) and 4% (third vs. fourth wave) increase, respectively. Moreover, the increased number of screening tests may be related to a lower test positivity rate in the later waves. This can be attributed to the increased testing capacity in the DRC, owing to the decentralisation of COVID-19 PCR laboratories and the use of Ag RDTs [31–33]. These testing capacity-building strategies are likely to have a more critical effect on the laboratory than the testing capacity itself. However, this testing capacity is not yet optimal, as we are still registering less than the recommended minimum of 10 tests per 10,000 inhabitants per week or 140 tests per day per million inhabitants [34].

The proportion of individuals with positive results and asymptomatic status, though not statistically significant, was greater at diagnosis during the second wave than during the first wave. This finding is a reminder of the trend of a decreasing number of symptomatic cases during the second wave that was observed in Nigeria [15]. A Spanish study showed that the proportion of patients who experienced severe clinical symptoms (defined as fever, cough, headache, dyspnoea, anosmia, ageusia and/or malaise for ≥ 4 days) was significantly lower during the second wave and, therefore, did not account for the proportion of individuals with minor symptoms that lasted ≤ 3 days [35]. We observed a significant decrease in CFR from 5.1% during the first wave to as low as 0.9% during the fourth wave. Immunity developed from natural immunity and vaccination, coupled with improved clinical care for COVID-19 patients, could explain this significant reduction. Omicron, which is strongly linked to the fourth wave in Congo, has been shown to cause less severe disease, lower hospitalisation rates, and significantly lower morbidity and mortality [32, 36].

The number of incident cases was greater during the waves of December and January compared to the waves in June, whilst the opposite was true for the number of deaths.

The COVID-19 pandemic might be contrasted with the 2009 H1N1 influenza pandemic, which demonstrated two specific waves throughout a calendar year. These influenza cases are still recorded during the seasons, including in outbreak forms [18]. The COVID-19 waves also appeared to follow this seasonal pattern, with the first and third wave happening about the same time—similarly with the second and fourth wave. This suggests the influence of ecological factors on the waves, in addition to the factors that have already been reported previously [18, 37]. Ecological factors are thought to influence the risk of exposure, and so modulate the effects on new populations, variants or reinfections. In addition, the seasonal patterns of infectious diseases and activity wave-forms are influenced by changes in social or prevention behaviours. The out-of-season outbreaks of the respiratory syncytial virus show a different trend compared with previous trends after the easing of COVID-19 physical distancing measures and were reported in France, Israel, Japan and Australia, among other countries [38–41].

From December to March, seasonal coronaviruses demonstrate a distinctly high point. Several factors affect the seasonality of specific diseases [18, 42–44]. This might include greater humidity associated with climate or patterns of social mixing. Some scientists project that COVID-19 will ultimately turn into a seasonal phenomenon similar to other types of coronaviruses [18, 45–47]. Additionally, seasonal patterns and wave formats of activity are heavily influenced by the quality of the immune response. The more immunity people develop towards a targeted pathogen, the lower the dynamics of transmission and general infection level, leading to a decline in spread and the emergence of new cases. Currently, the USA is far from developing collective immunity among most public groups. Nevertheless, a recent mathematical projection suggests that 43–60% of people should develop strong immunity against SARS-CoV-2 to gain the necessary positive effect [48].

The classical concept of herd immunity may not apply to COVID-19. Similar to the influenza virus, SARS-CoV-2 mutates continually into new variants that can escape immunity derived from infections and vaccines. Additionally, any level of herd protection against SARS-CoV-2 can be overcome by waning of infection- or vaccine-induced immunity. Thus, it is improbable that infection or vaccination will induce prolonged protection against SARS-CoV-2 [49]. However, a recent study suggested that by 31 December 2022, 43.5% of the DRC population would have had immunity (largely driven by natural immunity), following infections during the fourth wave, whereas only 0.1% of the population would have received a vaccine [50]. This is just within the proportion of the population needed for a positive effect. In 2022, the subsequent waves, together with increasing vaccination coverage, should increase this proportion further.

At present, the analysis that we have presented is based on reported data and may represent under-reporting. However, the information collated is crucial for building mathematical models to predict future patterns. The reductions in wave duration coupled with increased intensity in the presence of a new variant, together with the noticeable socio-ecological effects that influence the timing of waves, provide valuable information that can be incorporated into mathematical models to predict future patterns, after adjusting for the severity/infectiousness of different variants.

## Study Limitations

A limitation of our study was the use of the national testing line list which had some missing data, and it is possible that not all people who were tested had their data entered into the line list, and this may impact generalisability. However, the large amount of data that was analysed reduced the likelihood of bias that may arise due to missing data. Additionally, the information was primarily based on reported data, which was influenced by the testing strategies and capacities. However, detailed analysis across the African region has shown that even after correcting for under-reporting on the reporting date, the patterns/waves persisted in the reported data [50].

## Conclusion

This study provides a nationwide analysis of the epidemiological and clinical outcome characteristics of all four COVID-19 waves, which shows that increasing the SARS-CoV-2 testing capacity could lead to a significant decrease in the TPR and CFR. Each wave was associated with a particular SARS-CoV-2 variant and, when testing was coupled with continuous genomic surveillance, helped to keep the response system informed of the emergence or spread of any new variant of concern and its sublineages. Further analysis will help ascertain the major risk factors for SARS-CoV-2 transmission including importation and circulation of new variant of concern, morbidity including hospitalization and admission in intensive care units, and mortality in the DRC. Enhanced monitoring of COVID-19 waves of resurgence, increased testing capacity, sustained risk communication, improved clinical management capacity, genomic surveillance, and vaccination, especially of vulnerable populations, are measures that must be implemented and maintained to better control potential waves of COVID-19 resurgence in future outbreaks.

## Data Availability

Data are available on reasonable request.

## Abbreviations

Ag-RDT: Antigen rapid diagnostic test
CFR: Case fatality rate
CMR COVID-19: Comité Multisectoriel de la Riposte à la COVID-19 (in French) or Multi-sectorial Committee of the COVID-19 Response
COVAX: COVID-19 Vaccines Global Access
COVID-19: Coronavirus disease 2019
DRC: Democratic Republic of the Congo
EW: Epidemiological week
NPI: Non-pharmacological interventions
PCR: Polymerase chain reaction
SARS-CoV-2: Severe acute respiratory syndrome coronavirus 2
TPR: Test positivity rate
WHO: World Health Organization

## Declarations

### Provenance and peer review

Not commissioned, externally peer reviewed.
